# Design of High-Sensitivity Flexible Low-Profile Spiral Antenna Sensor for GIS Built-in PD Detection

**DOI:** 10.3390/s23104722

**Published:** 2023-05-13

**Authors:** Xukun Hu, Guozhi Zhang, Xiaotian Liu, Kang Chen, Xiaoxing Zhang

**Affiliations:** 1 Hubei Engineering Research Center for Safety Monitoring of New Energy and Power Grid Equipment, Hubei University of Technology, Wuhan 430068, China; 2010221106@hbut.edu.cn (X.H.); 102010278@hbut.edu.cn (K.C.); zhangxx@hbut.edu.cn (X.Z.); 2 Wuhan NARI Co., Ltd. of State Grid Electric Power Research Institute, Wuhan 430073, China; 3 School of Power and Mechanical Engineering, Wuhan University, Wuhan 430072, China; 4 Detroit Green Industry Institute, Hubei University of Technology, Wuhan 430068, China; 2011621109@hbut.edu.cn; 5 Xiangyang Industrial Institute, Hubei University of Technology, Xiangyang 441100, China

**Keywords:** GIS, PD, UHF, flexible antenna sensor, low profile

## Abstract

Spiral antenna sensors are commonly used in partial discharge (PD) ultra-high frequency (UHF) detection in gas-insulated switchgears (GISs). However, most of the existing UHF spiral antenna sensors are based on a rigid base and balun, such as FR-4. The safe built-in installation of antenna sensors requires the complex structural transformation of GISs. To solve this problem, a low-profile spiral antenna sensor is designed based on a polyimide (PI) flexible base, and its performance is optimized by improving the clearance ratio. The simulation and measurement results show that the profile height and diameter of the designed antenna sensor is 0.3 mm and 137 mm, which is 99.7% and 25.4% smaller than the traditional spiral antenna. Under a different bending radius, the antenna sensor can maintain VSWR ≤ 5 in 650 MHz~3 GHz, and its maximum *g*_re_ is up to 6.1 dB. Finally, the PD detection performance of the antenna sensor is carried out on a real 220 kV GIS. The results show that, after being built in, the PD with a weak discharge magnitude of 4.5 pC can be effectively detected by the antenna sensor, and the antenna sensor has the ability to quantify the severity of PD. In addition, through the simulation, the antenna sensor has potential for the detection of micro water in GISs.

## 1. Introduction

Gas insulated switchgears (GISs) are the core equipment of the power grid. If they fail, it may lead to serious safety accidents or economic losses [1,2]. Therefore, it is of great significance to accurately monitor their operation state. Partial discharge (PD) is a destructive electrical phenomenon caused by the extreme concentration of the partial electric field inside or on the surface of the insulator. PD can accelerate the deterioration of the insulation system, which may lead to the insulation breakdown of GISs [3]. When PD occurs, an ultra-high frequency (UHF, 300 MHz~3 GHz) signal can be excited and radiate around. The UHF method is used to detect PD by receiving this high-frequency electromagnetic wave through the antenna sensor. It has the advantages of non-contact, a strong anti-jamming ability, high sensitivity, positioning, and the pattern recognition of insulation defects [4,5]. Therefore, it has been widely used in PD detection of GISs [6].

The installation methods of UHF antenna sensors for GIS PD detection can be divided into three types: built-in, external, and dielectric windows [7,8]. In domestic settings, the installation methods are mainly concentrated in the built-in and external categories. The external UHF antenna sensor is used to detect PD UHF signal propagating outwardly from the pouring port on the GIS basin-type insulator. It is flexible in installation and use, but the UHF signal radiated outward through the pouring port attenuates it seriously. Moreover, it is easily interfered with through corona discharge and by communication signals from nearby equipment [9,10]. While the built-in UHF method is to directly install the antenna sensor into the GIS, this is not affected by the propagation attenuation of the PD UHF signal and external interference signal. It has the advantages of high sensitivity and an anti-jamming ability [11,12]. GISs produced in Britain must be installed with the built-in UHF detection system before delivery [13]. However, GISs have a compact structure with the development trend of miniaturization [14]. Spiral antenna sensors have the features of being frequency-independent, of high efficiency, and of good directivity in a wide frequency band, and are widely used in PD detection. When a spiral UHF antenna sensor is built into a GIS, it is affected by its rigid base and balun [15,16,17,18], and the GIS shell needs to be transformed to an outward convex structure to weaken the influence of the antenna sensor on the internal electric field distribution of the GIS [19]. Considering the conformal between the antenna sensor and GIS shell, Ref [20] designed a flexible spiral antenna sensor for GIS PD detection. However, the use of a traditional stereoscopic Balun leads to its profile height of 70 mm, which is not conducive to built-in installation. Therefore, built-in UHF detection systems are mostly installed before delivery. In conclusion, in order to expand the application of the built-in UHF method, it is urgent to develop a built-in spiral UHF antenna sensor with the advantages of flexible installation, high sensitivity, and with no impact on the normal operation of the GIS.

In view of the above problems, based on the flexible sensing technology, a study of the design and optimization of the built-in UHF antenna sensor based on the flexible base is carried out in this paper. A low-profile spiral antenna sensor is designed based on the flexible base. Then, its performance is optimized. Finally, the practical performance of the antenna sensor is tested by the vector network analyzer and the GIS PD detection experiment.

## 2. Antenna Sensor Design

### 2.1. Basic Principle of the Spiral Antenna Sensor

The Archimedes spiral antenna sensor is widely used in the fields of radar, satellite communication, and in condition monitoring of power equipment [21]. The parametric equation of the two-arm Archimedes spiral is as follows:

(1) $$\left\{ \begin{array}{l} r=r_{o}+a\varphi \\ r=r_{o}+a\left(\varphi -\pi \right) \end{array}\right.$$

where *r*_o_ is the starting radius, *a* is the spiral growth rate, and φ is the polar angle.

**Figure 1:**
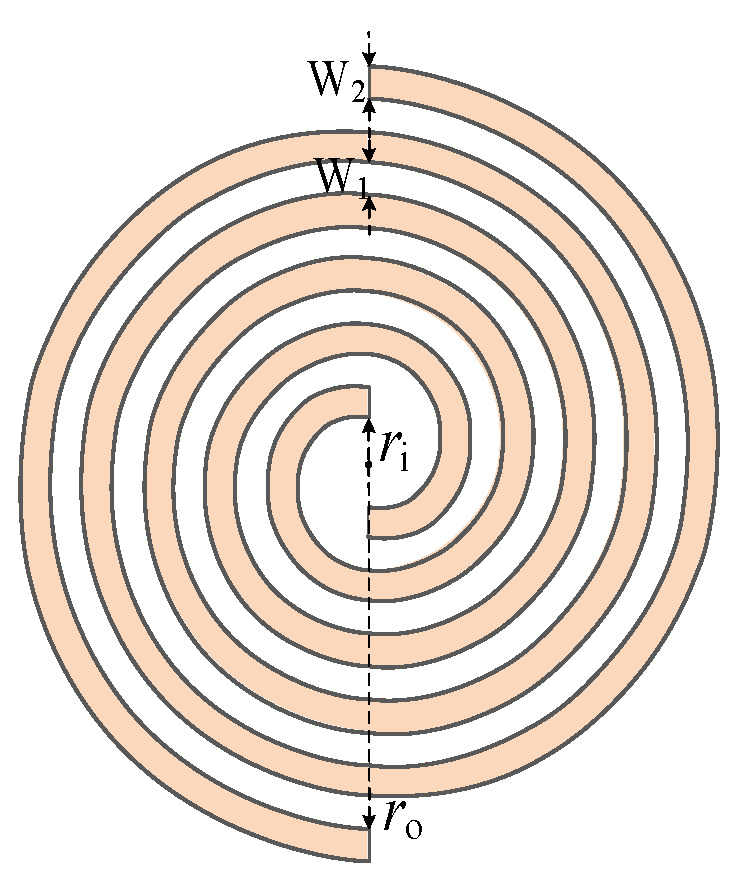
Structure diagram of Archimedes spiral antenna sensor.

Figure 1 is the structure of the Archimedes spiral antenna sensor. A self-complementary structure is formed when the arm width (W_2_) is equal to the gap width (W_1_) between the two arms. According to the Babinet principle [22], the structure can ensure the impedance stability of the antenna sensor in a wide frequency band, which is conducive to impedance matching and improves radiation efficiency.

The inner diameter *r*_i_ of the Archimedes spiral antenna sensor affects its maximum working frequency, and the value can be determined by the Formula (2). In general, *r*_0_ needs to be considered together with the size of the feeding device, which needs to cover the highest working frequency:

(2) $$2r_{i}\leq \lambda_{H}/4$$

where *λ*_H_ is the wavelength of the highest frequency.

The outer diameter *r*_o_ (the distance from the origin to the end of the antenna sensor arm) affects the minimum working frequency of the antenna sensor, which is generally taken as:

(3) $$2\pi r_{o}\geq 1.25\lambda_{L}$$

The UHF band is 300 MHz~3 GHz. When the minimum operating frequency of the antenna sensor is 300 MHz, according to formula (3), *r*_o_ is up to 199 mm. Too large a size in the antenna sensor is not conducive to the built-in installation and application of a GIS. Therefore, when designing a GIS built-in PD detection antenna sensor, it is particularly important to choose the appropriate working frequency band and structure.

### 2.2. Flexible Base

At home and abroad, a variety of flexible antenna sensors based on different flexible bases have been designed with the development of flexible materials [23,24,25,26]. Table 1 shows the comparison of the basic electrical parameters of PI, PDMS, PET, and FR-4 [27]. Among them, Polyimide (PI) is a commonly used flexible base called the “gold film”. In terms of physical properties, compared with FR-4, PI has the advantages of a light weight, good ductility, and deformability. In terms of electrical properties, the relative dielectric constant (*ε_r_*), dielectric loss factor (tanδ), and breakdown strength of PI are also better than that of FR-4. Signal delay and loss can be reduced under low *ε_r_* and tanδ, while high breakdown strength is also a major advantage of PI for the design of a GIS built-in PD detection antenna sensor. However, the flexible antenna sensors studied at present are mostly used in the field of communication, are wearable, and are rarely involved in the PD detection of GISs.

In view of the excellent performance of the PI flexible base, it is selected as the flexible base to design the built-in UHF antenna sensor in this paper, in order to solve the problems mentioned in the introduction.

### 2.3. Design and Optimization of the Flexible Low-Profile Spiral Antenna Sensor

**Figure 2:**
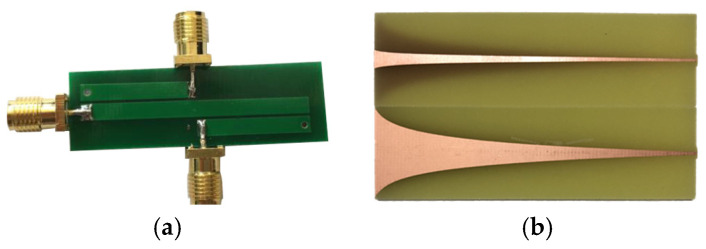
Traditional rigid base Balun. (**a**) Marchand Balun; (**b**) exponential gradient microstrip Balun.

Balun is a common method to realize a transformation in impedance for the self-complementary Archimedes spiral antenna sensor. As shown in Figure 2, the commonly used Balun are Marchand Balun [28], exponential gradient microstrip Balun [29], etc. However, the application of traditional stereoscopic Balun will increase the profile height of the spiral antenna sensor, which is not conducive to the GIS built-in inzstallation and application.

#### 2.3.1. Impedance Matching Based on Equiangular Spiral

**Figure 3:**
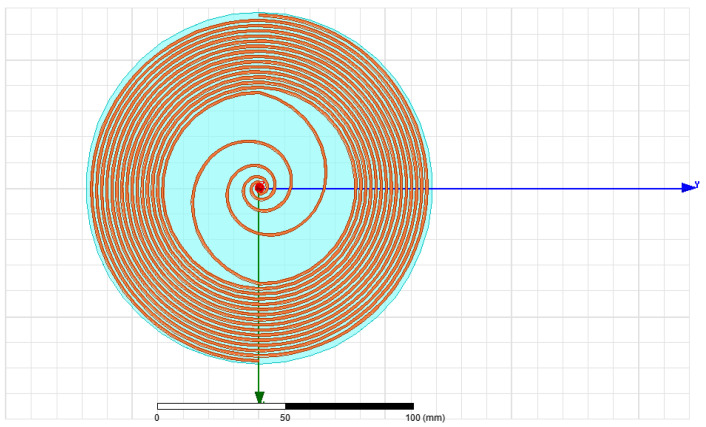
Impedance optimized 3D model based on equiangular spiral.

The polar radius of the equiangular spiral changes gently with the angle. It has the capability of impedance transformation and current chopping, similar to the exponential gradient microstrip Balun [30]. Therefore, in this paper, the impedance transformation structure based on the equiangular spiral is introduced to optimize the impedance matching of the self-complementary Archimedes spiral antenna sensor. The parametric equation of the antenna sensor structure is shown in Formula (4), one arm is obtained by rotating the other arm 180°. The 3D simulation model is shown in Figure 3:

(4) $$r=\left\{ \begin{array}{l} r_{1}e^{b\varphi }\left(0\leq \varphi \leq \varphi_{1}\right)\\ (r_{2}+c\varphi )\sin\left(\varphi \right)\left(0\leq \varphi \leq \varphi_{2}\right) \end{array}\right.$$

where *r*_1_ is the starting radius of the equiangular spiral, *r*_2_ is the starting radius of the Archimedes spiral, and *b* and *c* are the spiral growth rates of the equiangular spiral and Archimedes spiral, respectively.

**Figure 4:**
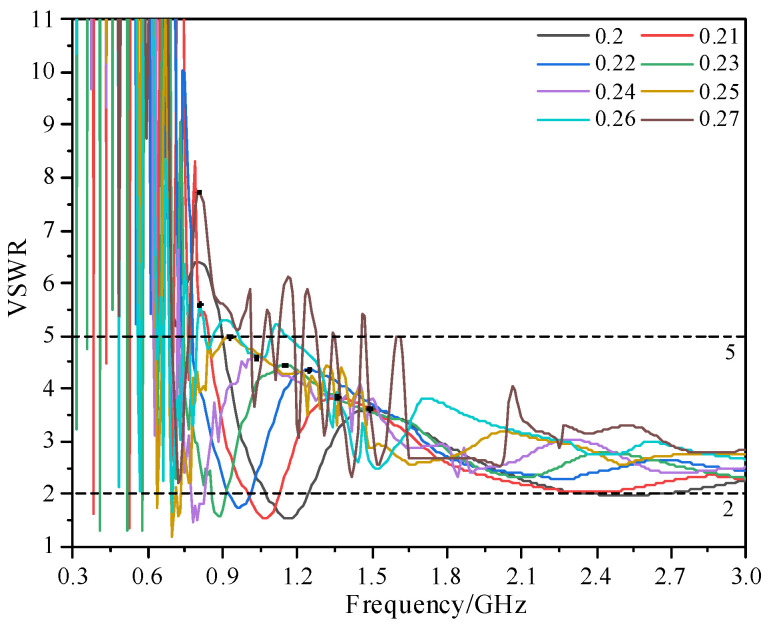
VSWR under different *b*.

Figure 4 shows the voltage standing wave ratio (VSWR) of the antenna sensor under different *b* when the W_2_ of the spiral is 1 mm, $\varphi_{1}$ is 4 π, *c* is 0.637, and $\varphi_{2}$ is changed to keep *r*_o_ constant. According to Figure 4, with an increase in *b*, the high-frequency performance of the antenna sensor is sacrificed to compensate for the low-frequency performance. However, when *b* is greater than 0.25, the VSWR of the antenna sensor has no obvious improvement. In addition, the bandwidth of VSWR ≤ 5 is close when *b* is 0.24 and 0.25. When *b* is too large, this will reduce the effective radiation area of the antenna sensor. Finally, *b* is set as 0.24 for the subsequent optimization.

It Is worth noting that, for the limited Internal space of a GIS, the built-in UHF antenna sensor is close to the PD source. Therefore, the electromagnetic energy received by the antenna sensor is still very strong when the VSWR is 5 (55.6% electromagnetic energy can be detected) [13]. Therefore, in this paper, the frequency band with VSWR ≤ 5 is defined as the effective frequency band of the antenna sensor.

#### 2.3.2. Effect of Clearance Ratio on Impedance Matching

The clearance ratio (*CR*) is the ratio of the arm width of the Archimedean spiral antenna sensor and the spacing between the two arms. According to Figure 1, *CR* can be defined as Formula (5):

(5) $$CR=\frac{W_{2}}{W_{1}}$$

**Figure 5:**
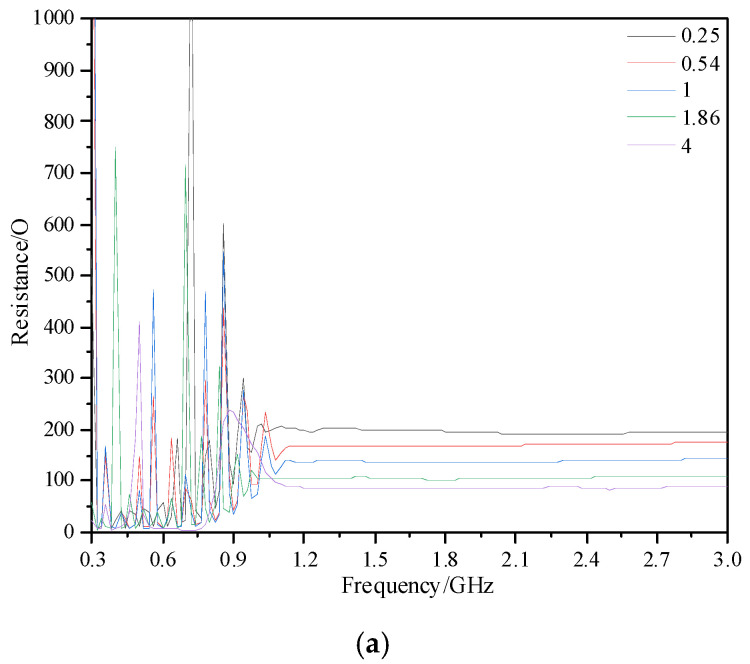

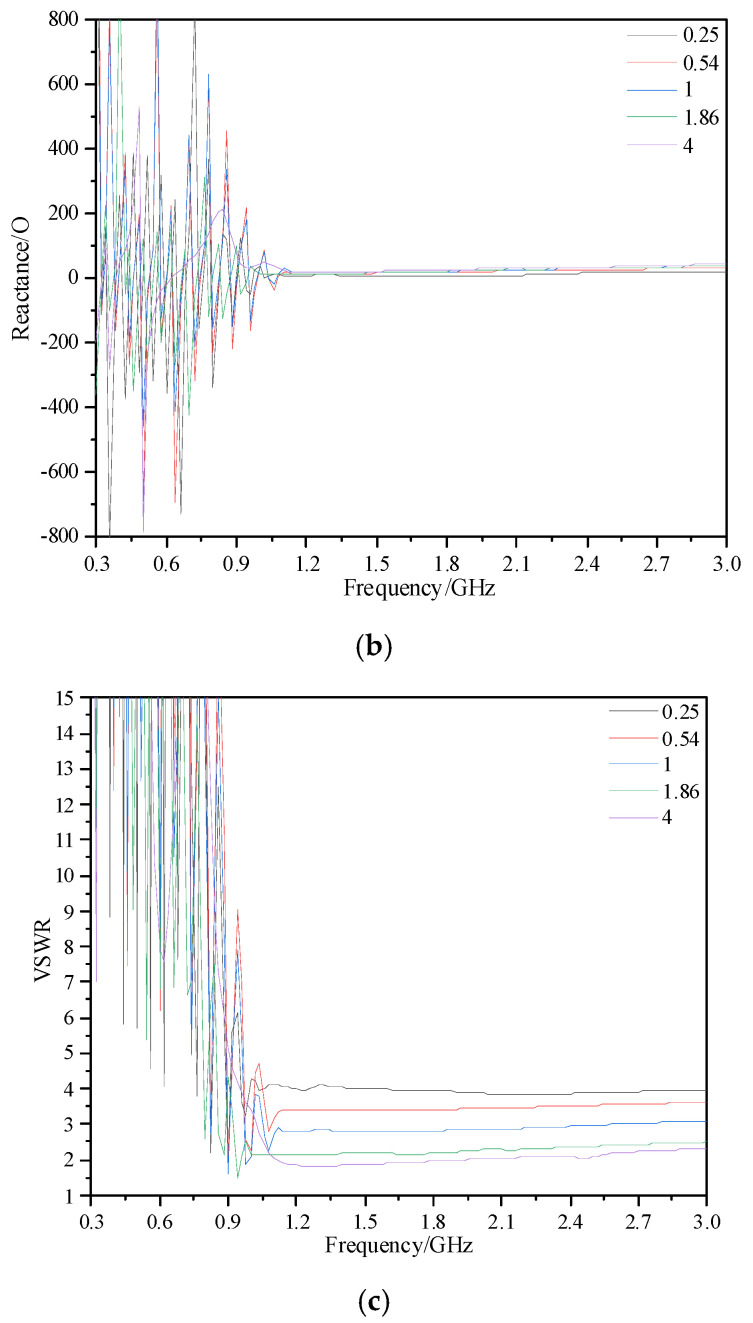
Variation of characteristic parameters of spiral antenna sensors under different *CR*. (**a**) Impedance; (**b**) reactance; (**c**) VSWR.

Figure 5 shows the impedance, reactance, and VSWR of the Archimedes spiral antenna sensor under different *CR*. In the high-frequency band (1 GHz~3 GHz), the impedance of the antenna sensor is steadily reduced from 195 Ω to 85 Ω with increasing *CR*, while the reactance is increased from 15 Ω to 35 Ω on average. The increase in reactance is not conducive to impedance matching, but the decrease in impedance is more obvious. On the whole, increasing *CR* is conducive to the impedance matching of the antenna sensor, which can be seen in Figure 5c. In addition, in the low-frequency band (300 MHz~1 GHz), the impedance can also be optimized with a suitable *CR*.

#### 2.3.3. Impedance Matching Optimization

Depending on the defect type, position, and shape of the GIS, there will be attenuation in the propagation of the PD UHF signals [31,32]. Therefore, it is necessary to further optimize the performance of the antenna sensor in the UHF frequency band to detect more GIS PD electromagnetic energy.

In Section 2.3.1, the equiangular spiral structure is introduced to optimize the VSWR of the Archimedes spiral antenna sensor in the low-frequency band, but the high-frequency performance is sacrificed. While, in Section 2.3.2, the VSWR is improved in the high-frequency band, in the low-frequency band, the oscillation of the VSWR is too large and the effective frequency band is narrow. Therefore, the VSWR of the antenna sensor is optimized based on the equiangular spiral structure and the *CR* in this paper. The specific method is to set the starting arm width as W_2_ + *l* (W_2_ = 1 mm), and linearly reduce its arm width in the inner ring (0~2 π). When φ is 2 π, the arm width becomes 1 mm, as shown in Formula (6). The *CR* of the inner ring of the antenna sensor can be effectively improved by this method.

(6) $$W=W_{2}+(-l/2\pi )\varphi +l\left(0\leq \varphi \leq 2\pi \right)$$

**Figure 6:**
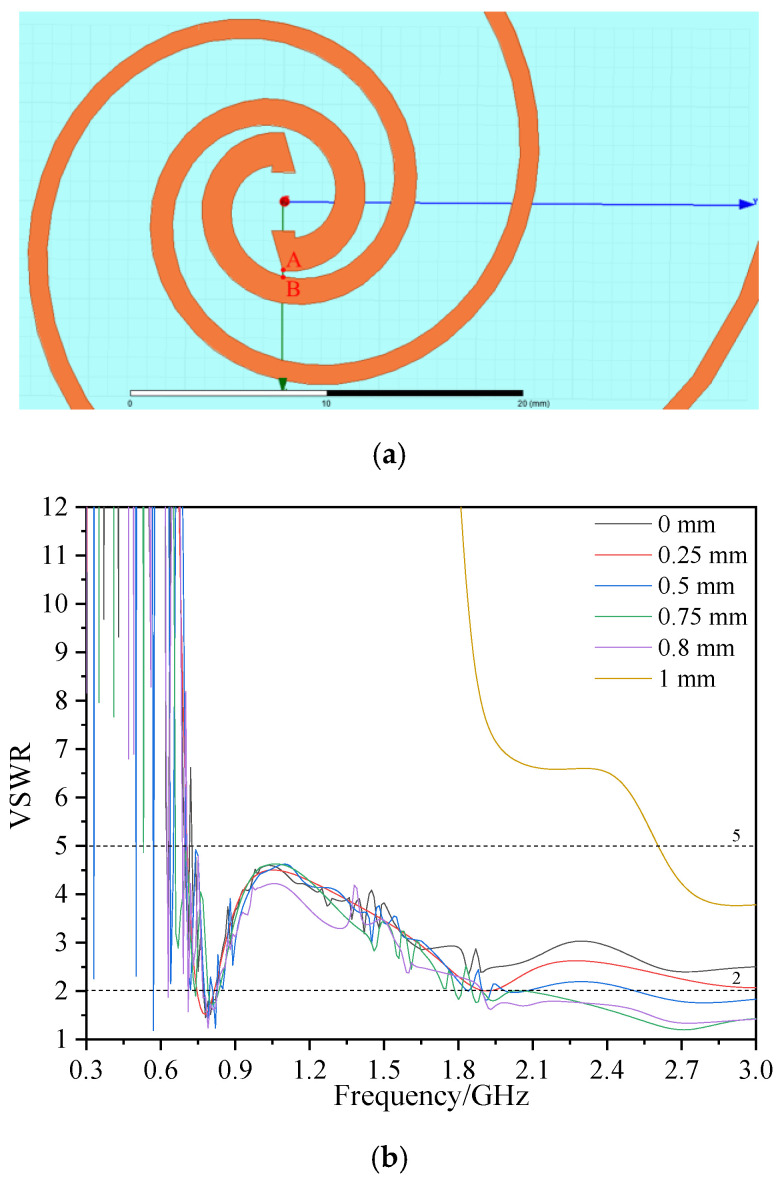
Optimized antenna sensor model and VSWR comparison. (**a**) Optimized structure of the equiangular spiral part of the antenna sensor; (**b**) VSWR under different *l*.

Figure 6a shows the structure of the equiangular spiral part after optimization, and the VSWR of the antenna sensor with different *l* is shown in Figure 5b. It can be seen that, under different *l*, the lowest frequency points at which the antenna sensor maintains VSWR ≤ 5 are 730 MHz, 710 MHz, 700 MHz, 660 MHz, 710 MHz, and 2.61 GHz, respectively. When *l* is in the range of 0~0.75 mm, the VSWR of the antenna sensor in the high-frequency band decreases with the increase in *l*. When *l* = 0.75 mm, the VSWR of the antenna sensor in the 1.85~3 GHz is ≤2. When *l* = 0.8 mm, the VSWR in the high-frequency band is similar to that of *l* = 0.75 mm, but the effective frequency band is not as good as *l* = 0.75 mm. According to Formulas (7)~(8), when *l* is too large, the spacing between point A and B in Figure 6a is too small and the line width increases, which leads to a sharp decrease in the distributed capacitance (*C*) and a sharp increase in the distributed inductance (*L*) of the antenna sensor. This will cause an impedance mismatch of the antenna sensor, and can be seen from the VSWR when *l* = 1 mm. Therefore, the final selected *l* is 0.75 mm.

(7) $$C=\frac{\varepsilon \pi }{\ln\frac{2W_{1}}{W_{2}}}$$

(8) $$L=\frac{\mu }{\pi }\ln\frac{2W_{1}}{W_{2}}$$

where ε is the dielectric constant and μ is the magnetic conductivity.

#### 2.3.4. Influence of Micro Water on Antenna Sensor Performance and Object Production

**Figure 7:**
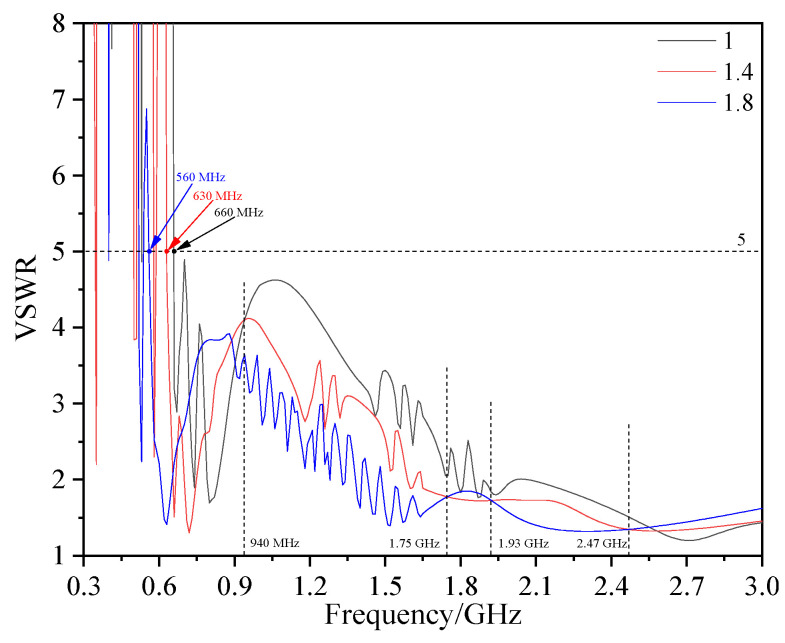
VSWR of the antenna sensor under different *ε*_r_.

If the insulating gas contains micro water, it will reduce the electrical strength and accelerate the breakdown of the insulation [33]. Therefore, in addition to insulation defects, micro water is also an important factor threatening the safe operation of GISs. Water is a strongly polar molecule, in which *ε*_r_ is up to 81. The change in trace moisture in insulating gas (*ε*_r_ = 1) will have a great influence on its *ε*_r_. Figure 7 shows the VSWR of the antenna sensor under different *ε*_r_ (*ε*_r_ increases with an increase in micro water). With the increase in *ε*_r_, the frequency point with VSWR ≤ 5 of the antenna sensor decreases from 660 MHz to 560 MHz. Moreover, in 940 MHz~1.75 GHz and 1.93~2.47 GHz, the VSWR decreases significantly with the increase in *ε*_r_. According to formulas (9) and (10), when *ε*_r_ increases, the propagation speed *v* of the electromagnetic wave slows down, so the wavelength *λ* under the same *f* decreases. Furthermore, according to formulas (2) and (3), the antenna sensor will have a better performance. In general, the VSWR of the antenna sensor in the insulating gas with different *ε*_r_ changes regularly, which has potential for the detection of micro water in GIS.

(9) $$v=\frac{1}{\sqrt{\mu \varepsilon }}$$

(10) $$\lambda =\frac{v}{f}$$

where, μ and ε are the magnetic conductivity and dielectric constant of the medium, respectively.

**Figure 8:**
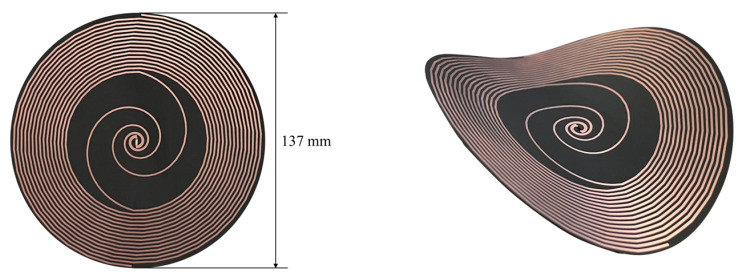
Object of flexible low-profile spiral antenna sensor.

The definitive object of the flexible low-profile spiral antenna sensor is shown in Figure 8. The diameter is 137 mm, which is 25.4% smaller than traditional spiral antenna. The thickness is only 0.3 mm, which is also its profile height. The profile height of the designed antenna sensor is reduced by 99.7% compared with the traditional spiral antenna sensor. In addition, in order to avoid the direct contact of the antenna sensor with insulating gas, the surface of the antenna sensor is coated with a layer of epoxy resin.

## 3. Antenna Sensor Performance Analysis

### 3.1. Voltage Standing Wave Ratio

**Figure 9:**
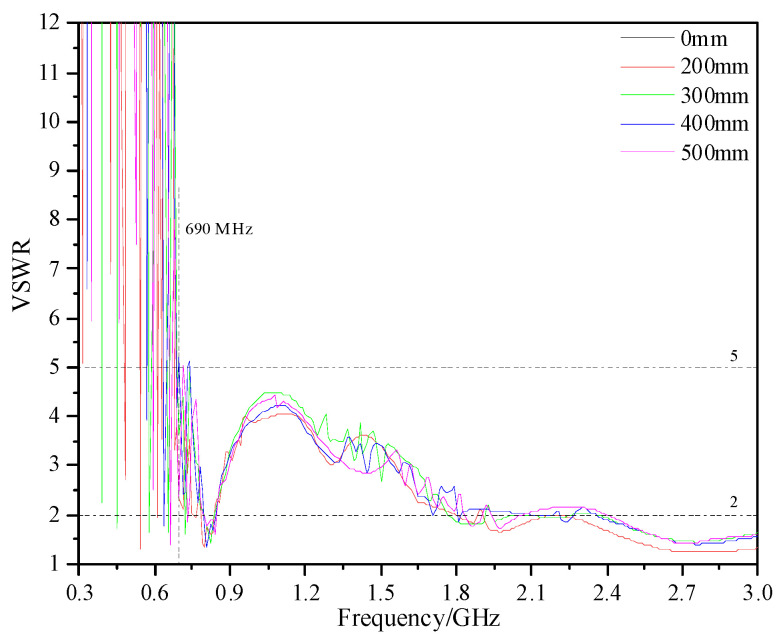
VSWR of simulation.

**Figure 10:**
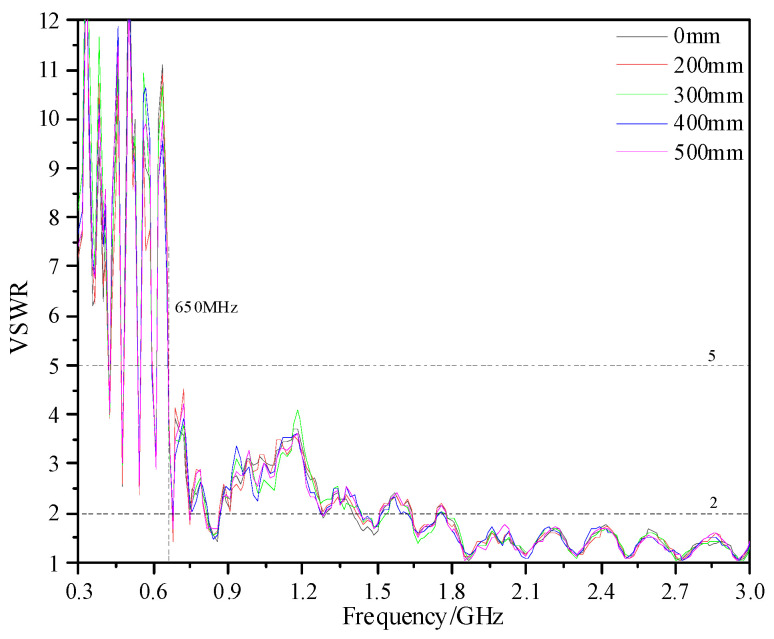
VSWR of measurement.

The shell of real GISs is a mostly tubular structure. Its bending radius is generally between 200 and 500 mm according to the different voltage level and manufacturing process. Therefore, in this paper, the performance of the designed antenna sensor under different bending radii (0, 200, 300, 400, and 500 mm) in 300 MHz~3 GHz is analyzed. The simulated VSWR is shown in Figure 9. Under different bending radii, the antenna sensor can keep VSWR ≤ 5 at 690 MHz~3 GHz and ≤2 at 2.4 GHz~3 GHz. The measured VSWR is shown In Figure 10, and the measuring equipment is an E5080A ENA vector network analyzer. The results show that, under different bending radii, the VSWR is ≤5 in 650 MHz~3 GHz, and the VSWR of some frequency points in 300 MHz~650 MHz is also ≤5. Moreover, the VSWR is ≤2 in 1.7 GHz~3 GHz and the average value is below 1.5. According to the simulation and measured results, the bending deformation does not affect the VSWR performance of the antenna.

Due to the diversity of errors (the convergence conditions, mesh, and characteristic impedance of the SMA adapter and coaxial line, etc.), there are some differences between the simulated and measured VSWR of the antenna sensor. However, the effective frequency band and variation trend of the VSWR are basically consistent. The effective frequency band can cover the main energy distribution frequency band of PD; in addition, the measured VSWR of the antenna sensor is ≤3.5 (70% electromagnetic energy can be detected) within 86% of the UHF band, which is an acceptable result [7].

### 3.2. Radiation Pattern

**Figure 11:**
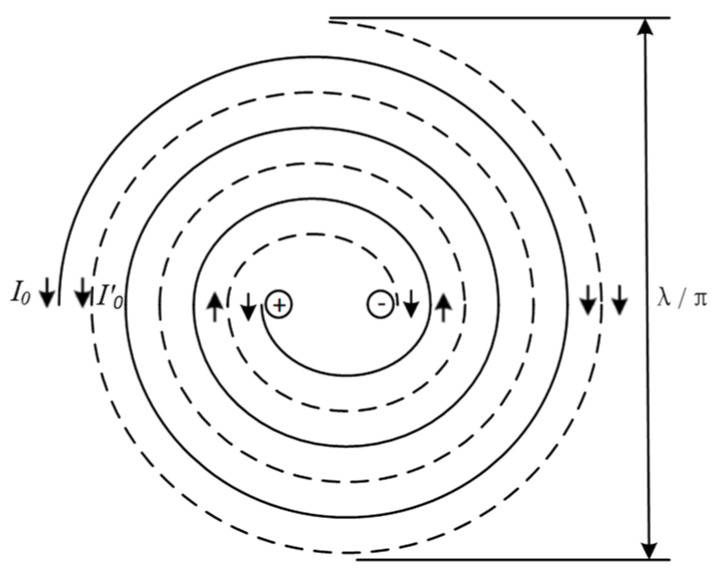
Radiation principle of self-complementary spiral antenna sensor.

Radiation patterns can represent the relative radiation intensity of the antenna sensor in different directions in space, and the directivity can be seen from it. As shown in Figure 11, the self-complementary spiral antenna sensor is a dual-arm structure with rotational symmetry. When an anti-phase feed is applied at the beginning of the antenna sensor arms, one arm is left-handed circular polarization and the other is right-handed circular polarization, which can form bidirectional radiation.

In this paper, the radiation intensity of the radiation pattern is described by the realized gain (*g*_re_), which represents the theoretical gain (*g*_th_) that takes into account the reflection loss and feeder loss. The relationship between *g*_re_, *g*_th_, and *S*_11_ can be expressed by Formula (11):

(11) $$g_{\mathrm{re}}=g_{\mathrm{th}}\left(1-S_{11}^{2}\right)$$

and the relationship between VSWR and *S*_11_ is as follows:

(12) $$\mathrm{VSWR}=\frac{1+\left|S_{11}\right|}{1-\left|S_{11}\right|}$$

**Figure 12:**
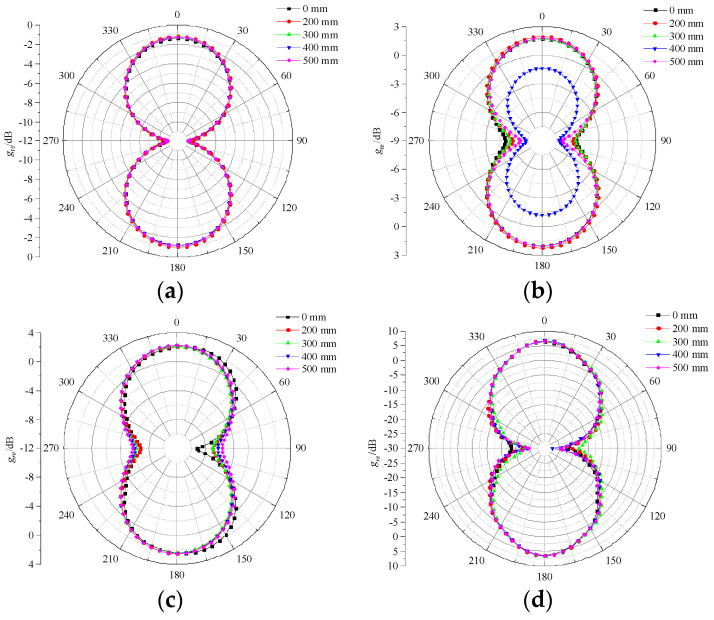
E-plane patterns at different frequency points. (**a**) 700 MHz; (**b**) 1 GHz; (**c**) 1.5 GHz; (**d**) 3 GHz.

**Figure 13:**
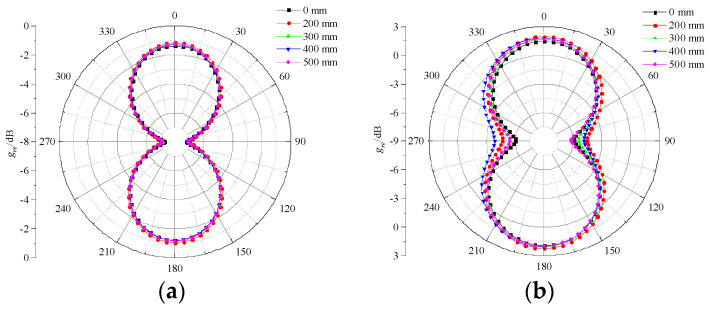

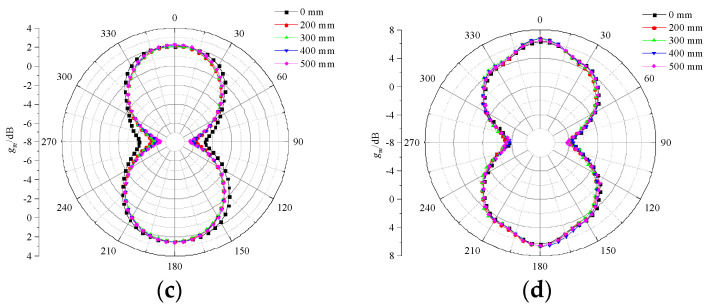
H-plane patterns at different frequency points. (**a**) 700 MHz; (**b**) 1 GHz; (**c**) 1.5 GHz; (**d**) 3 GHz.

Figure 12 and Figure 13 show the E-plane pattern and H-plane pattern of the designed antenna sensor under different bending radii. It can be seen that, under four frequency points (700 MHz, 1 GHz, 1.5 GHz, and 3 GHz), the E-plane and H-plane patterns of the antenna sensor are in good symmetry and directivity with the shape of “8”, which can receive PD UHF signals in all directions. Overall, under the same bending radius with different frequency points, the *g*_re_ of the antenna sensor tends to increase with an increase in frequency. The *g*_re_ under five bending radii increases from −1.5 dB at 700 MHz to 6.1 dB at 3 GHz. In addition, under the same frequency point with a different bending radius, the bending deformation has no obvious effect on the radiation features of the antenna sensor on the whole. However, when the bending radius is 400 mm, the radiation features of the E-plane deteriorate to a certain extent at 1 GHz, while having no effect on the H-plane.

In general, the flexible low-profile spiral antenna sensor designed in this paper has a good radiation performance. The *g*_re_ at 3 GHz is up to 6.1 dB. The higher *g*_re_ is more sensitive to a weak PD signal, which is conducive to the GIS PD detection.

## 4. PD Detection Performance Test

### 4.1. PD Detection Test Platform

**Figure 14:**
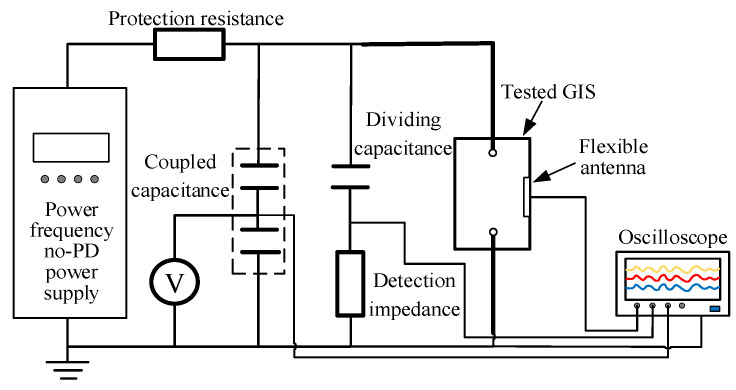
PD test platform.

In order to verify the PD detection performance of the designed flexible built-in low-profile spiral antenna sensor, in this paper, a PD test platform was set up, which was shown in Figure 14. The circuit meets the requirements of IEC 60270: 2015 [34]. Tektronix * MS044 high-performance digital oscilloscope (Sampling rate: 6.25 GS/s, Four channels) was used for signal acquisition. UHF signal, power frequency signal, and pulse current signal were collected by the three channels, respectively.

**Figure 15:**
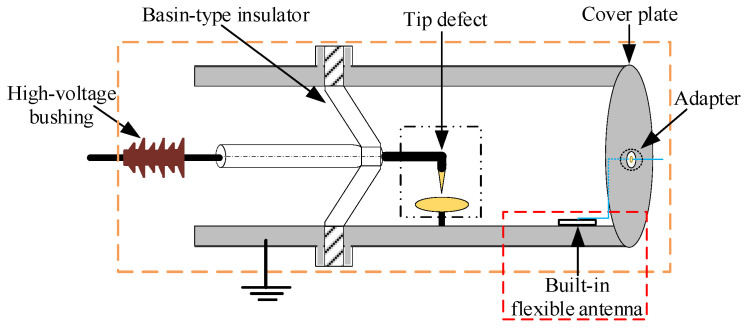
Tested GIS.

**Figure 16:**
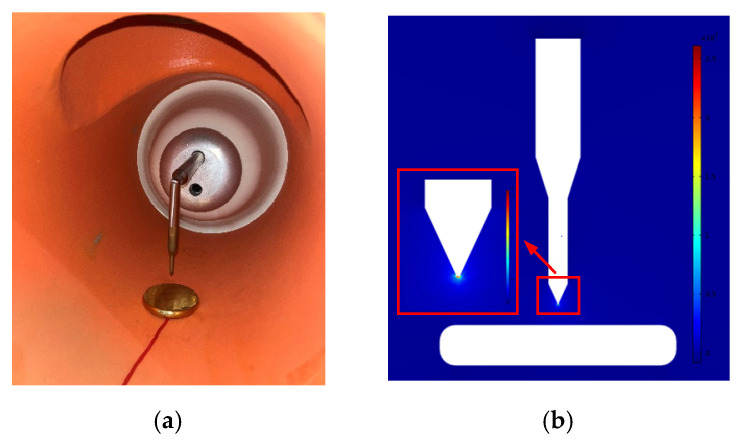
Tip defect. (**a**) Defect setting; (**b**) electric field distribution in tip gap.

The test was carried out on a real 220 kV GIS filled with 0.5 MPa SF_6_ insulating gas, as shown in Figure 15. The defect setting was shown in Figure 16a. The material of the electrode was brass. The needle electrode with a tip radius of 0.2 mm was directly connected with the high-voltage bus on the basin-type insulator. The grounding of the plate electrode was led out through the common grounding of the GIS shell, with a radius of 30 mm and thickness of 10 mm. The gap between the needle electrode and the plate electrode was 10 mm, which could be achieved by adjusting the height of the plate. Figure 16b shows the electric field distribution of the needle-plate electrode simulated by COMSOL. The field uniformity factor (*f*), computed using formula (13), was 28.83, which belonged to an extremely non-uniform electric field.

(13) $$f=\frac{E_{\max}}{E_{\mathrm{av}}}$$

where *E*_max_ and *E*_av_ were the maximum and average field strengths in the needle-plate electrode gap, respectively.

In terms of the installation of the flexible antenna sensor, first, the two arms of the antenna sensor were welded with the core and the grounding shield layer of the 50 Ω SMA coaxial line, respectively. The influence of the SMA-KE adapter on the GIS built-in installation of the antenna sensor could be avoided in this way. Then, after the artificial bending of the antenna sensor, a small amount of epoxy resin glue was used to make it perfectly conformal with the GIS shell. The bending radius of the GIS shell where the antenna sensor is placed was 200 mm, and the antenna sensor was 1 m away from the needle-plate defect. Finally, the PD UHF signal detected by the antenna sensor was led to the integration interface through the SMA coaxial line, so that we could receive PD signals outside the GIS.

**Figure 17:**
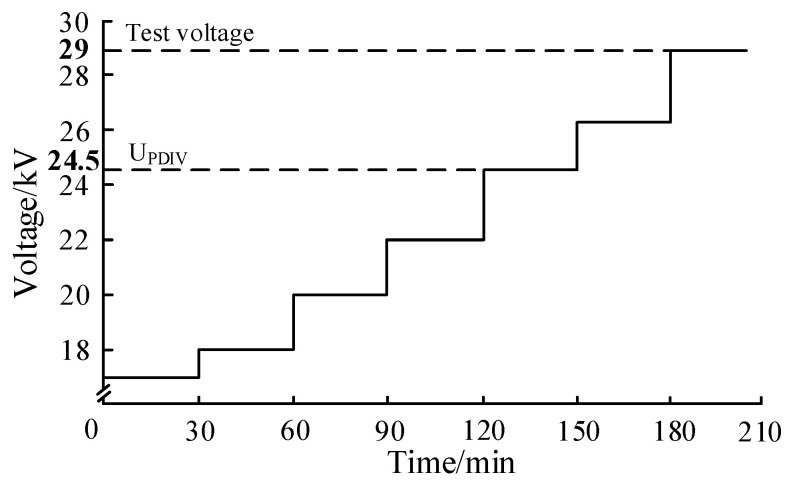
Step-stress method.

According to the DL/T 1432.4-2017 [35], the antenna sensor for the GIS PD detection test needs to be carried out under a discharge magnitude of 20 pC. Therefore, before the test, a PD discharge magnitude calibrator was used to calibrate the test platform. When the calibrated discharge magnitude was 20 pC, the peak of the pulse current signal was 14.6 mV. Furthermore, the step-stress method was used in the PD test, as shown in Figure 17. The PD inception voltage (U_PDIV_) of the platform was tested to be 24.5 kV.

### 4.2. GIS Built-in PD Detection Test

**Figure 18:**
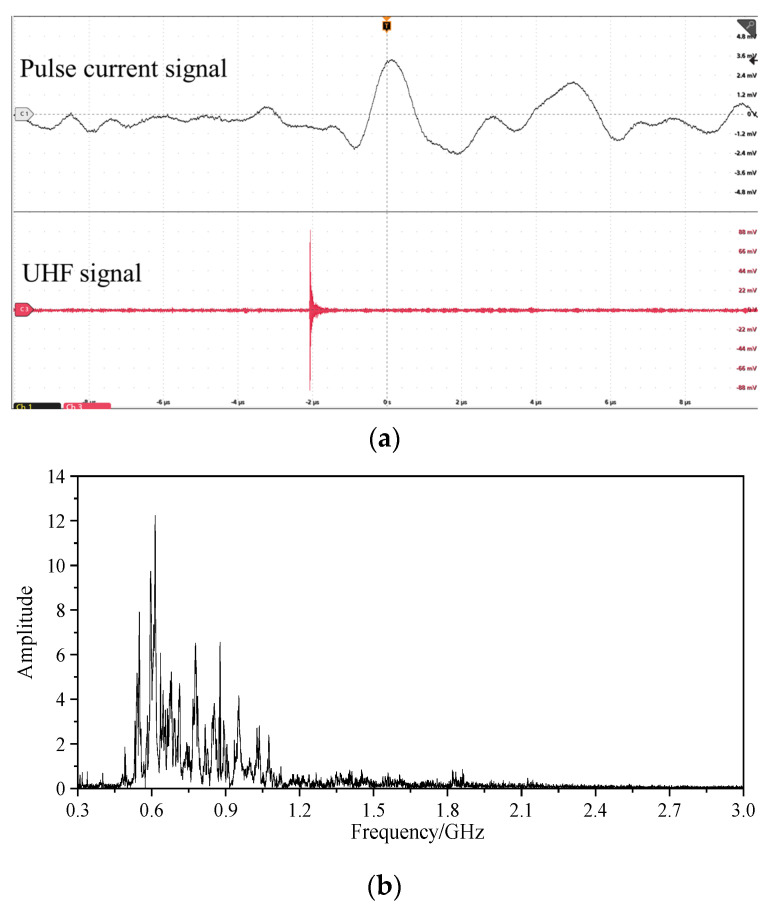
Single-pulse PD signal and UHF FFT analysis. (**a**) Single-pulse PD signal; (**b**) UHF FFT analysis.

When the test voltage was 29 kV, Figure 18a shows the UHF signal collected by the antenna sensor. It could be seen that the peak of the UHF signal was 90.2 mV under the discharge magnitude of 4.5 pC, which reflected the sensitivity of the high *g*_re_ to the weak PD signal. Moreover, the UHF signal could reflect the occurrence of PD together with the pulse current signal. Figure 18b shows the FFT analysis of the UHF signal of the tip discharge. Under these test conditions, the spectrum energy of the UHF signal was concentrated in 550 MHz~1 GHz and peaked at 620 MHz. The designed flexible antenna could effectively detect PD UHF signals in frequency bands with poor VSWR, and the built-in detection method was not subject to external electromagnetic interference.

**Figure 19:**
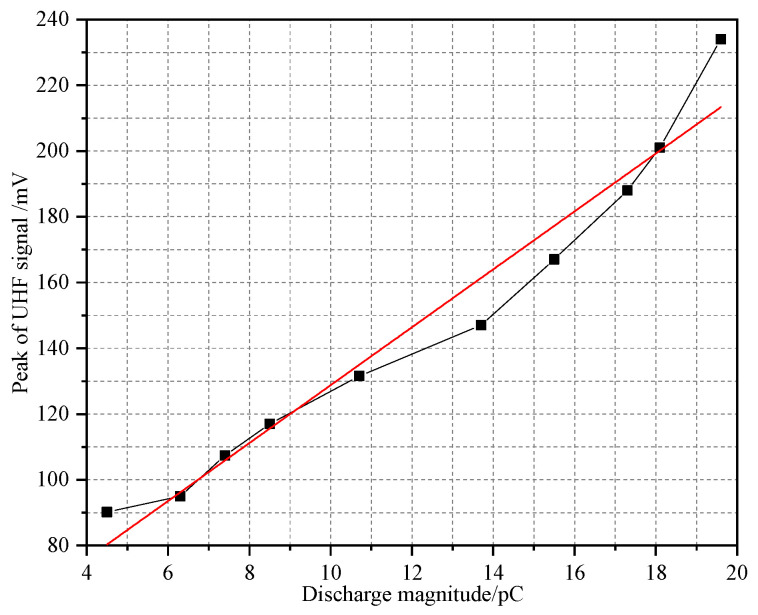
Peak statistics of UHF signal under different discharge magnitudes.

Figure 19 was the peak statistics of UHF signal under the discharge magnitude ≤20 pC. It is obvious that the peak of the UHF signal obtained by the antenna sensor increased as the discharge magnitude increased, with a high linearity. After linear fitting, the *R^2^* reached 0.958, which had the ability to quantify the severity of PD while achieving GIS insulation state monitoring.

**Figure 20:**
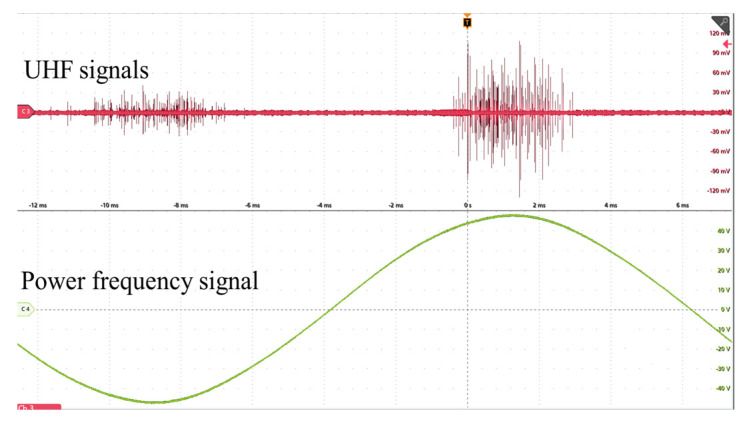
PD UHF signal in one power frequency period.

Figure 20 shows the phase spectrum information of the UHF signal of the tip discharge in one power frequency period (20 ms), and Ch4 was the power frequency signal. The UHF signal pulses mainly occurred between 60° and 120° in the positive half cycle of power frequency, and were less distributed in the negative half cycle, with obvious phase features. This was conducive to subsequent fault recognition based on the PD UHF signal. The antenna sensor could accurately capture the PD UHF signals, and the test results further verified that the antenna sensor met the requirements of the GIS built-in PD detection.

## 5. Summary

In view of the shortcomings of the existing antenna sensor for GIS built-in PD detection, in this paper, PI is introduced to design a UHF flexible low-profile spiral antenna sensor. Then, the radiation performance of the antenna sensor is analyzed by finite element simulation and measurement. Finally, the PD detection performance of the antenna sensor is verified by the GIS PD test platform. The conclusions are as follows:

(1) The simulation and measurement results show that, the low-frequency and high-frequency performance of the antenna sensor is optimized by improving the inner ring *CR* of the equiangular spiral. In total, 89.1% of the UHF band can be covered with VSWR ≤ 5. The maximum *g*_re_ under different bending radii is up to 6.1 dB. The diameter and profile height of the designed antenna sensor is 137 mm and 0.3 mm, which is 25.4% and 99.7% smaller than the traditional spiral antenna. These features provide conditions for the antenna sensor to be flexibly built into GISs.
(2) The VSWR of the antenna sensor in the insulating gas with different *ε*_r_ changes regularly. Therefore, it has the potential for the detection of micro water in GISs.
(3) The GIS built-in PD test shows that the low discharge magnitude of the PD signal (4.5 pC) could be accurately detected by the antenna sensor. In addition, the antenna sensor had the ability to quantify the severity of PD while achieving GIS insulation state monitoring.

## Figures and Tables

**Table 1:** Comparison of basic electrical parameters of FR-4/PI/PDMS/PET base.

	Material	FR-4	PI	PDMS	PET
Parameter					
*ε_r_*		4.4	3.5	3	4
tanδ		0.026	0.004	0.02	0.04
Breakdown strength (normal temperature)/(kV∙mm^−1^)		40	180	20	380

## Data Availability

The data presented in the article is original and has not been inappropriately selected, manipulated, enhanced or fabricated by us.
